# Metadata Analysis of *mcr-1*-Bearing Plasmids Inspired by the Sequencing Evidence for Horizontal Transfer of Antibiotic Resistance Genes Between Polluted River and Wild Birds

**DOI:** 10.3389/fmicb.2020.00352

**Published:** 2020-03-10

**Authors:** Yufei Lin, Xiaohong Dong, Jiao Wu, Dawei Rao, Lihua Zhang, Yousef Faraj, Kun Yang

**Affiliations:** Department of Pharmaceutical and Biological Engineering, School of Chemical Engineering, Sichuan University, Chengdu, China

**Keywords:** antibiotic resistance, intercontinental dissemination, migratory birds, antibiotic resistance gene, horizontal gene transfer

## Abstract

We sequenced the whole genomes of three *mcr-1*-positive multidrug-resistant *E. coli* strains, which were previously isolated from the environment of egret habitat (polluted river) and egret feces. The results exhibit high correlation between antibiotic-resistant phenotype and genotype among the three strains. Most of the mobilized antibiotic resistance genes (ARGs) are distributed on plasmids in the forms of transposons or integrons. Multidrug-resistant (MDR) regions of high homology are detected on plasmids of different *E. coli* isolates. Therefore, horizontal transfer of resistance genes has facilitated the transmission of antibiotic resistance between the environmental and avian bacteria, and the transfer of ARGs have involved multiple embedded genetic levels (transposons, integrons, plasmids, and bacterial lineages). Inspired by this, systematic metadata analysis was performed for the available sequences of *mcr-1*-bearing plasmids. Among these plasmids, IncHI2 plasmids carry the most additional ARGs. The composition of these additional ARGs varies according to their geographical distribution. The phylogenetic reconstruction of IncI2 and IncX4 plasmids provides the evidence for their multiregional evolution. Phylogenetic analysis at the level of mobile genetic element (plasmid) provides important epidemiological information for the global dissemination of *mcr-1* gene. Highly homologous *mcr-1*-bearing IncI2 plasmids have been isolated from different regions along the East Asian-Australasian Flyway, suggesting that migratory birds may mediate the intercontinental transportation of ARGs.

## Introduction

Horizontal gene transfer (HGT) plays an important role in the global dissemination of antibiotic resistance, while the mobilization of ARGs is the first and also the most important evolutionary step for their horizontal transfer. The discovery of any new mobilized resistance gene always attracts great attention of the researchers. As evidenced by a number of studies, these mobilized resistance genes have been increasingly spreading all over the world (Stokes and Gillings, 2011). Typical cases include the discovery of *bla*_KPC_, *bla*_NDM_, and *mcr-1* genes. The *bla*_KPC_ gene was first identified in 1996, and now it can be detected in many regions of the world (Munoz-Price et al., 2013). Its mobilization is related to a 10 kb Tn*3*-based mobile transposon Tn4401 (Naas et al., 2008; Cuzon et al., 2011). The *bla*_NDM_ gene was first identified in 2008 in a *K. pneumoniae* isolate recovered from a Swedish patient, who had previously been hospitalized in New Delhi, India (Yong et al., 2009). Thereafter, this resistance gene was detected in different regions of the world (Dortet et al., 2014). Its mobilization is associated with an IS*Aba125* composite transposon Tn125 (Poirel et al., 2012). The *mcr-1* gene was first discovered in China (Liu et al., 2016), and the same ARG was then reported in various regions of the world (Wang et al., 2018). Its mobilization is mediated by an IS*Apl1* composite transposon Tn6330 (Snesrud et al., 2016; Li et al., 2017).

Although the effect of HGT on the dissemination of antibiotic resistance has been realized, the spread of specific resistant bacterial clones is also widely concerned (Munoz-Price et al., 2013; Matamoros et al., 2017). In early 1990s, it was found that the moving of transposons between carrier replicons (plasmids) resulted in the spread of ARGs between bacterial species (Liebert et al., 1999). Recently, it has been described as a nested Russian doll-like mobility of ARGs, which once again drew the attention of researchers (Sheppard et al., 2016; Wang et al., 2018; Hernando-Amado et al., 2019). These studies clearly explain the mechanism of the horizontal transfer of resistance genes at multiple genetic levels (transposons, integrons, plasmids, bacterial lineages and bacterial species) (Wang et al., 2018). In our previous study (Wu et al., 2018), we demonstrated that wild birds could transport antibiotic resistance from contaminated river to the surrounding environment, and the spread of antibiotic resistance was not mainly due to the transfer of resistant bacterial clones. Among those resistant *E. coli* isolates in the aforementioned work, no strong correlation was observed between strain genotypes (repetitive-element PCR genotyping) and their drug-resistance patterns. Therefore, we concluded that the horizontal transfer of resistance genes was the main mechanism of resistance transmission in that process. Nonetheless, there was no direct sequencing evidence at the time. In this study, we sequenced the whole genomes of three *mcr-1*-positive multidrug-resistant *E. coli* strains isolated in the previous work to pursue the evidence for horizontal transfer of resistance genes. Inspired by the sequencing results, we systematically analyzed *mcr-1*-bearing plasmid sequences downloaded from GenBank database. We tried to obtain epidemiological information of the *mcr-1* gene by analyzing its global dissemination at multiple levels of mobile genetic elements (MGEs), especially at the plasmid level.

## Materials and Methods

### *mcr-1*-Positive Strains

In our previous study, we found that the wild birds (egrets) could mediate the environmental transmission of antibiotic resistance in local area, from the polluted Jin River to birds’ nightly habitat, Wangjianglou Park (Wu et al., 2018). Some of the *E. coli* strains isolated in that work exhibited colistin resistance (*n* = 6), among which three isolates were proved to be *mcr-1*-positive and multidrug-resistant. Two of the three strains were isolated from the river (Jin River) polluted by antibiotic-resistant bacteria and the third one was isolated from egret feces. Three *E. coli* isolates were respectively labeled as W5-6, W2-5, and BE2-5.

### Antibiotic Susceptibility Testing

The minimum inhibitory concentrations (MICs) of 10 antibiotics against the three *E. coli* isolates has previously been determined (Wu et al., 2018) via a modified broth micro-dilution method as per ISO 20776-1:2006 CLSI using 96-well microtiter plates (Clinical and Laboratory Standards Institute [CLSI], 2019). The only modification in our method is in the step of inoculum preparation. Typically, the *E. coli* isolates were first grown in LB-broth-loaded 96 well plate at 37°C overnight to reach the stationary phase. The cell cultures were then 10^3^ fold diluted and used as inoculum. When inoculating the testing 96-well plates containing antibiotics of different concentrations, a 48-pin replicator was used to improve the efficiency of experimental operation. The MIC endpoints were determined as the lowest concentration, at which there was no visible growth after 20 h of incubation at 37°C. Duplicate tests for each antibiotic concentration were conducted. Positive and negative controls were conducted in antibiotic-free LB to ensure the growth of environmental *E. coli* isolates under lab conditions and sterility of the assay, respectively. Quality control of the procedure was conducted by using the susceptive *E. coli* standard strain ATCC 25922.

### Bacteria Culture and Whole Genomic DNA Extraction

The whole genome DNA was extracted from fresh bacterial cell mass recovered from 100 mL LB pure culture containing colistin of 4 μg/mL with the FastDNA^®^ Spin Kit for Soil (MP Biomedicals, France). About 1.2 mL of high-quality DNA was obtained for each *E. coli* strain. A small amount of each DNA sample was used for the amplification and sequencing of 16S rRNA gene to ensure that the DNA sample was obtained from a pure culture. The presence of *mcr-1* gene in the DNA sample was also verified via PCR. The primer pair for amplifying 16S rRNA gene was 27F (5′-AGAGTTTGATCCTGGCTCAG-3′) and 1522R (5′-AAGGAGGTGATCCANCCRCA-3′) and that for *mcr-1* was CLR5-F (5′-CGGTCAGTCCGTTTGTTC-3′) and CLR5-R (5′-CTTGGTCGGTCTGTAGGG-3′) (Liu et al., 2016). Thereafter, the remaining bacterial DNA samples were kept frozen (−40°C) until sequencing.

### Whole Genome Sequencing for Three *E. coli* Isolates

The bacterial genome DNA was detected by agarose gel electrophoresis and quantified by Qubit. The genomes of the three isolates were sequenced using Single Molecule, Real-Time (SMRT) technology performed at Beijing Novogene Bioinformatics Technology Co., Ltd. The low quality reads were filtered by the SMRT 2.3.0 (Berlin et al., 2015), and the filtered reads were *de novo* assembled to generate contigs without gaps (Koren and Phillippy, 2015). All genome sequences (chromosome and plasmid sequences) of the three *E. coli* isolates were deposited into GenBank database under the BioProject PRJNA495707 with BioSample numbers SAMN10230266 to SAMN10230268 and accession numbers CP032986 to CP032995.

The strain types (STs) of the three *E. coli* isolates were determined from their assembled genomes using online MLST service provided by the Center for Genomic Epidemiology^1^ according to Achtman’s MLST scheme (Wirth et al., 2006; Larsen et al., 2012).

### Prediction of Open Reading Frames (ORFs) and Gene Functions

We used GeneMarks to predict ORFs for the three *E. coli* isolates (Besemer et al., 2001). We used seven databases to predict gene functions. They were GO (Gene Ontology) (Ashburner et al., 2000), KEGG (Kyoto Encyclopedia of Genes and Genomes) (Kanehisa et al., 2004, 2006), COG (Clusters of Orthologous Groups) (Tatusov et al., 2003), NR (Non-Redundant Protein Database) (Li et al., 2002), TCDB (Transporter Classification Database) (Saier et al., 2014), Swiss-Prot (Bairoch and Apweiler, 2000), and TrEMBL (Magrane and UniProt, 2011), respectively. A whole genome BLAST search (*E*-value less than 10^–5^, minimal alignment length percentage larger than 40%) was performed against above seven databases (Altschul et al., 1990). ARGs were annotated via BLAST searching the ORFs against the Comprehensive Antibiotic Resistance Database (CARD) (Liu and Pop, 2009; McArthur et al., 2013). We set the thresholds of *e*-value and “best identity” at 10^–5^ and 80%, respectively.

### Epidemiological and Phylogenetic Analysis of the *mcr-1*-Bearing Plasmids

The discovery of the mobilized colistin resistance gene, *mcr-1*, is in the era of rapid development of next-generation sequencing technology. During only 4 years, hundreds of sequences of *mcr-1*-bearing plasmids have been uploaded to the database, which provides a good chance for metadata analysis of these plasmids. We retrieved the GenBank database with keywords “***mcr-1***” (or *mcr1*) and “**plasmid**” in June 2018. From the hit entries, the **circular** sequences were manually picked and recorded the metadata. Identification of the plasmid incompatibility group was performed for downloaded plasmid sequences via the CGE online services PlasmidFinder v1.3^2^ (Carattoli et al., 2014). ResFinder^3^ was used to determine other acquired resistance genes on these plasmids (Zankari et al., 2012).

Most of the published *mcr-1-*bearing plasmids belong to three main incompatibility groups, IncHI2, IncI2, and IncX4 (Li et al., 2017; Matamoros et al., 2017). According to the respective characteristics of the three incompatibility plasmid groups, we performed a targeted analysis. The analysis routine is illustrated in Supplementary Figure S1.

## Results and Discussion

### Genome Assembly and Annotation for Three *E. coli* Isolates

The original sequencing data obtain more than 50 × coverage of the whole genome for the three *E. coli* isolates. Chromosomes and plasmids of the three isolates are all assembled in circular contigs with no gaps. Three *E. coli* isolates are of different strain types (Table 1). The number, size and Inc-types of plasmids are also different among the three isolates (Table 1). *E. coli* W5-6 contains 3 plasmids. The other two isolates both carry 2 plasmids. The plasmid pMCR_W5-6 gives the biggest size (241 kbp) among all plasmids. The numbers of genes predicted by GeneMarks are 4898, 4834, and 4576 for W5-6, W2-5, and BE2-5, respectively. The annotation of the three genomes with the COG database clusters these genes into 23 classes with annotation rate of 89.6, 90.7, and 93.8%, respectively (Supplementary Figure S2). Genomes of all the three *E. coli* strains, especially the strain W5-6, contain a considerable number of MGEs (Mobilome in Supplementary Figure S2).

**TABLE 1:** Genome assembly results for three *E. coli* isolates.

Isolates	Type	Contig ID	Size (bp)	GC%	Circular?	ST or Inc^c^	Accession
W5-6	Chromosome	W5-6Chr	4,638,901	50.7	Circular	ST2	CP032992
	Plasmid	pMCR_W5-6^a,b^	241,043	46.42	Circular	IncHI2 (IncHI2A)	CP032993
	Plasmid	p2_W5-6	44,779	44.83	Circular	IncX1	CP032994
	Plasmid	p3_W5-6	72,717	51.52	Circular	IncN (IncFIA, IncFIB)	CP032995
W2-5	Chromosome	W2-5Chr	4,914,512	50.56	Circular	ST355	CP032989
	Plasmid	pMCR_W2-5^a^	66,380	42.92	Circular	IncI2	CP032990
	Plasmid	p2_W2-5	83,867	51.13	Circular	IncN (IncFII)	CP032991
BE2-5	Chromosome	BE2-5Chr	4,677,021	50.76	Circular	ST532	CP032986
	Plasmid	pMCR_BE2-5^a^	51,622	46.91	Circular	IncP1	CP032987
	Plasmid	p2_BE2-5^b^	84,688	50.74	Circular	IncR (IncX1)	CP032988

*^*a*^The plasmids bearing mcr-1 gene. ^*b*^The plasmids with MDR region of similar structure. ^*c*^Strain type (ST) or incompatibility replicon type of plasmids (Inc). Multiple replicons on the same plasmid are indicated in bracket.*

### Antibiotic-Resistant Phenotype and Genotype of Three *E. coli* Isolates

All the three strains show multidrug resistance. The *E. coli* W5-6 demonstrates the highest level of drug resistance among the three isolates. It shows resistance against 9 out of 10 tested antibiotics. The other two *E. coli* isolates of lower resistance levels are resistant to 7 antibiotics (Table 2).

**TABLE 2:** Antibiotic-resistant phenotype and corresponding resistance genes or genetic mutations in three *E. coli* isolates.

Isolates (Origin)	Phenotype and genotype	Aminoglycosides				Tetracyclines	Cephalosporins	Penicillins	Polypeptides	Quinolones	
		Kan	Ami	Gen	Str	Tet	Cef	Amp	Col	Cip	Nal
W5-6 (Jin River)	AR (MIC)	R (> 128)	S (8)	R (> 128)	R (> 128)	R (> 128)	R (> 6.4)	R (> 128)	R (> 16)	R (> 6.4)	R (> 128)
	Gene	*aph(3′)-Ia aph(4)-Ia*		*aac(3)-IV*	*aph(3″)-Ib aadA1 aadA2 aph(6)-Id*	*tet(B) tet(D)*	*bla*_CTX–M–__14_	*bla*_TEM–__1_ *bla*_CTX–M–__14_	*mcr-1*	*oqxA oqxB gyrA*^a^ *parC*^a^	*gyrA*^a^ *parC*^a^
W2-5 (Jin River)	AR (MIC)	S (16)	S (8)	S (4)	R (> 128)	R (64)	R (> 6.4)	R (> 128)	R (> 16)	R (> 6.4)	R (> 128)
	Gene				*aph(3″)-Ib aph(6)-Id*	*tet(A)*	*bla*_CTX–M–__55_	*bla*_CTX–M–__55_	*mcr-1*	*gyrA*^a^ *parC*^a^	*gyrA*^a^ *parC*^a^
BE2-5 (Egret feces)	AR (MIC)	R (> 128)	S (8)	S (8)	R (128)	R (> 128)	S (0.025)	R (> 128)	R (16)	R (1.6)	R (16)
	Gene	*aph(3′)-Ia*			*aadA1 aadA2*	*tet(A)*			*mcr-1*	*qnrS2*	*qnrS2*

*AR, Antibiotic resistance; MIC, Minimum inhibitory concentration, μg/mL; Kan, kanamycin A; Ami, amikacin; Gen, gentamicin; Str, streptomycin; Tet, tetracycline; Cef, ceftriaxone; Amp, ampicillin; Col, colistin; Cip, ciprofloxacin; Nal, nalidixic acid; S, means susceptible; R, means resistant. ^*a*^Point mutations in these genes confer resistance to fluoroquinolones. For specific detail, please refer to Table 3.*

Eighty-five putative ARGs (via BLAST against the CARD database) are annotated in the whole genome of *E. coli* W5-6, 20 of which are on plasmids. The numbers of ARGs on chromosomes of *E. coli* W2-5 and BE2-5 are 65 and 66, respectively. The numbers of ARGs on plasmids of these two isolates are 3 and 11, respectively (Table 3). Fifty-two ARGs are common among all the three *E. coli* isolates. However, most of these shared ARGs (*n* = 44, 84.6%) are efflux pump genes, and they are distributed on their chromosomes, which implies that efflux pump genes are highly conserved in the same bacterial species. These efflux pump genes do not appear to be significantly associated with the antibiotic-resistant phenotype of the three strains. Although these efflux pump genes may be just involved in some detoxification process (Martinez et al., 2009), we still list them below (Table 3) for reference. The only non-efflux-pump common ARG with a definite resistant phenotype among the three isolates is the colistin resistance gene *mcr-1*. The *mcr-1* gene is located on the plasmids of different Inc-types in the three *E. coli* strains. The plasmids pMCR_W5-6, pMCR_W2-5, and pMCR_BE2-5 are of the IncHI2, IncI2, and IncP1-type, respectively (Table 1). The *mcr-1* sequence on plasmid pMCR_W2-5 lost the downstream IS*Apl1* (Supplementary Figure S3).

**TABLE 3:** CARD database annotated ARGs in three *E. coli* isolates.

Isolates	Location	Resistance mechanism					
		Efflux pump	Antibiotic inactivation	Target replacement/protection	Altering cell wall charge	Gene variant/mutant	Others (molecular bypass, absence, modulating permeability etc.)
W5-6	Chromosome	A^a^, *acrF, mdtB, tet(B)*^*b*^, *tet(D)*^*b*^	*aph(3′)-Ia, aph(6)-Id aph(3″)-Ib, catI*	*mfd, sul2*	*arnA, pmrC, pmrE, pmrF*	*glpT* (E448K)^*c*^; *gyrA* (S83L, D87N)^*c*^; *parC* (A56T, S80I, A620V)^c^	*bacA, lamB*
	pMCR_W5-6	*cmlA1, floR, qacH, oqxA, oqxB*	*aac(3)-IV, aadA1, aadA2, aph(3′)-Ia, aph(4)-Ia, bla*_CTX–M–__14_,*fosA3*	*dfrA12, sul1, sul2, sul3*	*mcr-1*		
	p2_W5-6		*bla*_TEM–__1_				
	p3_W5-6	*floR, vgaC*					
W2-5	Chromosome	A^a^, *floR, mdtF*^*b*^, *muxB, tet(A)*	*aph(3″)-Ib, aph(6)-Id*	*mfd*^*b*^, *sul2*	*arnA, pmrC, pmrE, pmrF*	*glpT* (E448K)^*c*^; *gyrA* (S83L, D87N)^*c*^; *parC* (S80I)^*c*^	*bacA, lamB*
	pMCR_W2-5				*mcr-1*		
	p2_W2-5	*vgaC*	*bla*_CTX–M–__55_				
BE2-5	Chromosome	A^a^, *mdtF*^*b*^, *muxB, tet(A)*		*mfd*	a*rnA, pmrC, pmrE, pmrF*	*glpT* (E448K)^*c*^	*bacA, lamB*
	pMCR_BE2-5				*mcr-1*		
	p2_BE2-5	*cmlA1, mef(B), qacH, tet(A)*	*aadA1, aadA2, aph(3′)-Ia*	*dfrA12, qnrS2, sul3*			

*^*a*^A represents the common efflux pump genes in all three *E. coli* isolates, which include acrA, acrB, acrE, acrR, acrS, baeR, baeS, cpxA, cpxR, crp, emrA, emrB, emrD, emrE, emrK, emrR, emrY, evgA, evgS, gadW, gadX, hns, kdpE leuO, marA, mdfA, mdtA, mdtE, mdtF^*b*^, mdtG, mdtH, mdtL, mdtM, mdtN, mdtO, mdtP, mexN, msbA, msrB, patA, rob, tolC, yojI. ^*b*^There are two copies of mdtF on the chromosome of W5-6, three copies of mdtF on both chromosomes of W2-5 and BE2-5, two copies of mfd on the chromosome of W2-5, and two copies of tet(B) and tet(D) on the chromosome of W5-6. ^*c*^Specific point mutations in each gene are listed in the bracket. The reference sequences are from the genome of E. coli K-12 MG1655 (Accession No. NC_000913.3).*

For Aminoglycosides, resistance genes encoding three types of antibiotic inactivation enzymes are detected among the three *E. coli* isolates, aminoglycoside acetyltransferases [*aac(3)-IV*], phosphotransferase [*aph(3′)-Ia*, *aph(4)-Ia*, *aph(3″)-Ib* and *aph(6)-Id*] and nucleotidyltransferase (*aadA1* and *aadA2*) genes. *E. coli* W5-6 possesses all the three kinds of resistance genes (Table 2), and exhibits the highest resistance level to aminoglycosides. It is resistant to three out of four tested aminoglycosides: kanamycin A, gentamicin and streptomycin (Table 2). *E. coli* W2-5 has the ARGs, which render resistance to streptomycin [*aph(3″)-Ib* and *aph(6)-Id*], and exhibits resistance to this antibiotic. *E. coli* BE2-5 does not have these two ARGs, while it has *aph(3′)-Ia*, which enables it to resist against kanamycin A (McArthur et al., 2013). For tetracycline, we detected corresponding ARGs in the genomes of all the three *E. coli* isolates either on chromosome [*tet(A)*, *tet(B)* and *tet(D)*] or on plasmid [*tet(A)*], which is in good agreement with their resistance to this antibiotic (Tables 2, 3). For β-lactams, different β-lactamase genes are detected on the plasmids of *E. coli* W5-6 (*bla*_CTX–M–__14_ on pMCR_W5-6 and *bla*_TEM–__1_ on p2_W5-6) and W2-5 (*bla*_CTX–M–__55_ on p2_W2-5), while no β-lactamase gene is detected for *E. coli* BE2-5, as it is susceptible to ceftriaxone (Table 2). For quinolones, genes encoding subunits of efflux pump complex conferring resistance to fluoroquinolone (*oqxA* and *oqxB*) (Kim et al., 2009) are detected on the plasmid (pMCR_W5-6) of *E. coli* W5-6. Point mutations in *gyrA* and *parC* genes conferring resistance to fluoroquinolones are detected in *E. coli* W5-6 and W2-5 (Oram and Fisher, 1991; Tankovic et al., 1996). In *E. coli* BE2-5, *qnrS2* gene is detected on its plasmid (p2_BE2-5), which confers it weak resistance to ciprofloxacin and nalidixic acid (Gay et al., 2006; Table 2). Besides above ARGs conferring resistance to the tested antibiotics, resistance genes against sulfonamides (*sul2* etc.), chloramphenicol (*cmlA1*), fosfomycin (*fosA3*), and trimethoprim (*drfA12*) are also detected among the three *E. coli* isolates. Point mutation in *glpT* gene conferring resistance to fosfomycin is detected in all three *E. coli* isolates (Takahata et al., 2010; Table 3). Overall, the antibiotic-resistant genotype is in good agreement with the resistant phenotype among the three *E. coli* isolates. The only exception is that no corresponding resistance gene is detected to explain the unexpected resistance of *E. coli* BE2-5 against ampicillin.

### Evidence for the Horizontal Transfer of ARGs Between Environmental and Avian *E. coli*

**FIGURE 1:**
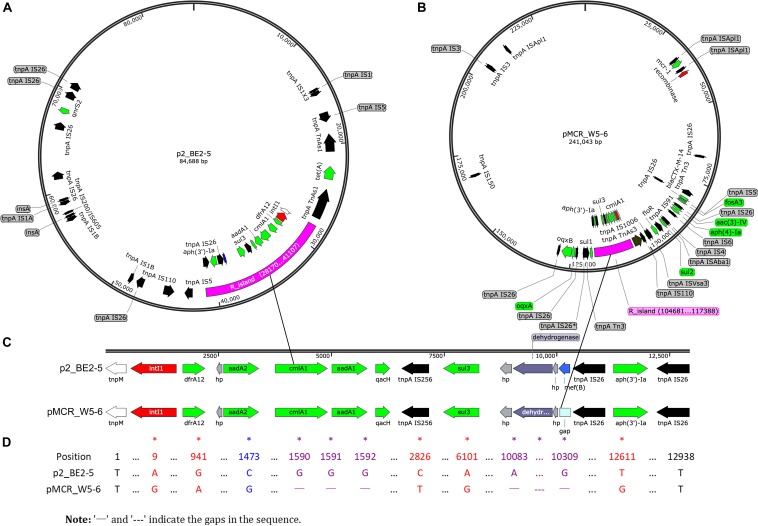
Sequencing evidence for horizontal transfer of ARGs between the environmental (polluted Jin River) and avian (egret) *E. coli*. **(A,B)** Exhibit the distribution of ARGs (green arrows) and MGEs on plasmids p2_BE2-5 and pMCR_W5-6, respectively. The MDR regions (R_island) of the similar structure are indicated as purple fragments on both plasmids. Their structures in detail are illustrated in **(C)**, and the sequence alignment of two R_islands is exhibited in **(D)**. The MDR region is composed of one integron (red arrows indicate site-specific integrase/recombinase genes) and one IS*26*_*aph(3′)-Ia*_IS*26* composite transposon (black arrows for transposase genes, *tnpA* IS*26* etc.). The recombination event (*mef(B)* gene) in the MDR region is indicated in blue (**A,C**, on plasmid p2_PE2-5). The mismatches between sequences were indicated with asterisks in **(D)**. A BRIG-Easyfig version of this figure is shown in supporting information as Supplementary Figure S4.

Almost all the ARGs detected in the avian *E. coli* (BE2-5) are found in two other environmental strains (W2-5 and W5-6, Table 3), which is consistent with our previous view that the antibiotic resistance (resistant bacteria or resistance genes) is mainly transferred from the polluted river (Jin River) to the wild bird (egret) (Wu et al., 2018). Most importantly, highly homologous MDR regions are detected on two plasmids of different Inc-types (pMCR_W5-6 and p2_BE2-5) in *E. coli* W5-6 and BE2-5, respectively (Figures 1A,B). The sizes of the MDR regions are around 12,800 bp (Figure 1D). Although the size and overall structure of the two plasmids are different (Figures 1A,B), the composition of the ARGs and MGE sequences (gene sequences encoding transposase and integrase) and their arrangement in the MDR regions are almost the same (Figure 1C and Supplementary Figure S4). The only difference is that the MDR region on the plasmid pMCR_W5-6 missing an efflux pump gene *mef(B)* in comparison to the plasmid p2_BE2-5. On the plasmid pMCR_W5-6, there even remains a 33 nt 3′ fragment of the *mef(B)* gene. This provides sequencing evidence for gene deletion and recombination during the evolution of the MDR region. The shared ARGs includes the genes conferring resistance to aminoglycosides [*aadA1*, *aadA2* and *aph(3′)-Ia*], sulfonamides (*sul3*), trimethoprim (*drfA12*) and chloramphenicols (*cmlA1*). The shared MGE sequences, especially the IS*26* sequences that are detected more than 5 copies on both plasmids (Figures 1A,B and Supplementary Figure S4), should play an important role during the formation of the MDR regions, and provide conditions for the relocation of these ARGs. Since the highly homologous MDR regions are detected on the plasmids of different Inc-types and in the *E. coli* of different strain types, the evolution of the MDR regions must have involved the process of HGT.

The identical drug-resistant transposon containing *aph(3′)-Ia* gene is also found on the chromosome (W5-6Chr) and plasmids (pMCR_W5-6 and p2_BE2-5) of different bacteria. Similarly, the resistance gene [*aph(3′)-Ia*] is also flanked with transposase genes (*tnpA* IS*26*) on both sides (Supplementary Figure S5). The ARGs on other plasmids are illustrated in Supplementary Figure S6. Most of these ARGs also show close correlation with MGEs.

The spread of antibiotic resistance is closely related to the horizontal transfer of resistance genes, regardless in the clinical or in a natural environment, which is highly polluted with antibiotics or antibiotic resistant bacteria (Stokes and Gillings, 2011). In our case, Jin River (an urban river in Chengdu, Sichuan, China) is highly polluted with antibiotic resistant bacteria, and the avian inhabitants (egrets) there are also highly affected. The resistance genes conferring antibiotic-resistant phenotype in the three *E. coli* isolates are mainly distributed on plasmids or on chromosome, but in forms of transposons (Supplementary Figure S5). The resistance genes on plasmids are also closely related to MGE sequences (transposase and integrase genes on plasmids as shown in Figure 1 and Supplementary Figure S6). From environmental (polluted river water) and avian (egret) *E. coli* of different strain types, we have detected almost identical MDR regions on the plasmids of different incompatibility types. ARGs, which are flanked by MGE sequences, present as transposons or integrons in the MDR regions. Identical drug-resistance transposon [IS*26*-*aph(3′)-Ia*-IS*26*] is also found on chromosome and plasmids of these host bacteria. These results indicate that HGT plays a crucial role in the environmental dissemination of antibiotic resistance, and the transfer of ARGs must involve multiple embedded genetic levels (transposons, integrons, plasmids, and bacterial lineages), which is so-called the nested “Russian doll” model of genetic mobility (Sheppard et al., 2016).

### Global Dissemination and Multiregional Evolution of *mcr-1* Plasmids

**FIGURE 2:**
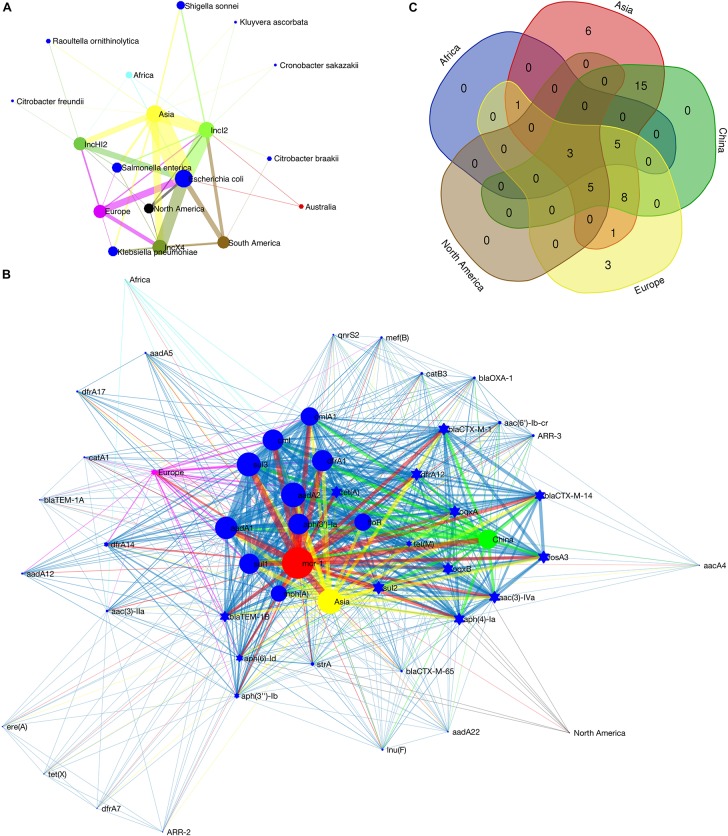
Detection rate of *mcr-1*-bearing plasmids in various bacterial host species on different continents **(A)**, co-occurrence network of *mcr-1* with additional ARGs on IncHI2 plasmids **(B)** and Venn diagram showing the numbers of shared and unique additional ARGs on IncHI2 plasmids at different regions **(C)**. In **(A)**, the size of the nodes (plasmid type, bacterial species or continent) is proportional to their detection rate (log-transformed), and the width of the edge between two nodes is proportional to the detection rate of one node (plasmid type or bacterial species) from another (bacterial species and/or continent). Blue nodes indicate bacterial species, green of different saturations the plasmid types and the nodes of other colors the continents. In **(B)**, the size of the nodes (ARG or geographical region) indicates the frequency of their occurrence, and the width of the edges is proportional to the co-occurrence rate between two nodes (between two ARGs or between an ARG and its location). Blue nodes indicate additional ARGs, red the *mcr-1* gene and the nodes of other colors the geographical regions. Ten most frequently occurred ARGs are shown as blue circles.

The metadata of 228 circular *mcr-1*-bearing plasmids were collected, as shown in Supplementary Table S1. The geographical distribution of plasmids of different incompatibility groups is depicted in Supplementary Figure S7. It is worth noting that the map outlines only the types of plasmid replicons on different continents. Some plasmids carry two or more (up to five) replicons (Supplementary Table S1). Therefore, a plasmid may have been counted more than once. The result still reflects the local diversity of plasmid types. Asia contributes the most types of *mcr-1*-bearing plasmids (13 out of 16). The other two continents in the Northern Hemisphere, Europe and North America also exhibit high diversity of plasmid types, 9 and 6, respectively. On the contrary, the regions in the Southern Hemisphere show poor plasmid diversity (Supplementary Figure S7). Sampling bias may have led to this discrepancy. However, twenty-eight *mcr-1*-bearing plasmids recovered from South America give only two plasmid types, IncX4 and IncI2. Up to some extent it reflects that the Southern Hemisphere (at least South America) suffers fewer invasions of various *mcr-1*-bearing plasmids. Among these *mcr-1-bearing* plasmids, the numbers of IncI2, IncX4 and IncHI2 plasmids are ranked at the top 3. Their detection rates in various *Enterobacteriaceae* hosts on different continents are illustrated in Figure 2A. IncI2 plasmids exhibit the most diverse bacterial hosts (in 6 unique host species of *Enterobacteriaceae*) and the most extensive geographical distribution (in all continents).

The IncHI2-type plasmid is the most diverse plasmid and harbors a large MDR region (Li et al., 2017). Therefore, it is considered as a genetic element mediating the transmission of MDR genes. A wide range of resistance genes and MGEs can be found in a Mosaic MDR region of IncHI2-type plasmids (Li et al., 2017). We recorded the additional ARGs on each IncHI2 plasmid (Supplementary Table S1). Co-occurrence network analysis, showing the correlation between these ARGs and their geographic distribution, was performed in Matlab_R2016a. The composition of ARGs in this MDR region varies according to their geographical distribution (Figure 2B). Such an MDR region is also discovered in our sequenced IncHI2-type plasmid, pMCR_W5-6 (Figure 1B). Unlike other ARGs concentrated in such an MDR region, *mcr-1* gene is located in another location on the plasmid away from the MDR region (Supplementary Table S1 also lists the interval between *mcr-1* and other ARGs). It has a unique transposon structure, IS*Apl1*-mcr-1-orf-IS*Apl1* (Snesrud et al., 2016; Li et al., 2017). The insertion of *mcr-1*-bearing transposon in such type of plasmid should be a late independent event. The exact time of the insertion event is hard to be determined, but the backbone structure of the IncHI2-type plasmid at the time of *mcr-1* insertion can be speculated. The ARGs with high occurrence frequency should be the original backbone structure of this type of plasmids (Figure 2B). Besides *mcr-1*, two other ARGs, *floR* and *aadA2*, are common all over the world on this type of plasmids. It has been reported that the resistance (*floR*) to florfenicol – a veterinary drug – is commonly associated with *mcr-1* (Matamoros et al., 2017; Shen et al., 2018), which supports the animal origin of the mobilized *mcr-1* gene (Matamoros et al., 2017; Wang et al., 2018). The *mcr-1*-bearing IncHI2 plasmids recovered from Asia (mainly from China) possess the most diverse ARGs (Figure 2C), which must be due to the extensive use of antibiotics in this area (Zhang et al., 2015). The three unique ARGs on European IncHI2 plasmids are *aadA12*, *bla*_TEM__1__A_ and *catA1*. This may be attributed to regional evolution events in Europe.

The IncX4-type plasmids are the most conserved and the smallest (mostly around 33 kb) ones among all types of *mcr-1*-bearing plasmids (Li et al., 2017). The sequences of IncX4-type plasmids were adjusted from the same start point and aligned with the multiple sequence alignment program MAFFT^4^ (Katoh et al., 2017). The aligned sequences were used to construct a phylogenetic tree in MEGA7 (Kumar et al., 2016). The IncI2-type plasmids contain a site-specific recombination system, the shufflon. The shufflon generates variants of the PilV protein, a minor component of the thin pilus. The shufflon is one of the most difficult regions for *de novo* genome assembly, because of its structural diversity even in a single bacterial colony (Sekizuka et al., 2017). Therefore, the shufflon structure affects the alignment of the plasmid sequences, and thus affects the phylogenetic reconstruction. For this type of plasmid (*n* = 92), we excised the shufflon region from each plasmid sequence and used the remaining part for phylogenetic analysis as we did for IncX4-type plasmids. From the phylogenetic trees of the two types of plasmid (Supplementary Figures S8, S9A), we detected the genotypic clusters of a single geographical origin. Typically, the plasmid clusters of South America are observed in both phylogenetic trees, which indicates relatively seldom exchange of *mcr-1*-bearing plasmids between South America and other geographical regions. Geographical barriers may result in such a regional evolution. Meanwhile, Asia shows the closest communication with other geographical regions, and the interconnection between Europe and North America is relatively high. In addition, according to the phylogenetic trees of the two types of plasmids, the intercontinental exchange of IncI2 plasmids seems more frequent than that of IncX4 plasmids. The diversity of bacterial hosts of IncI2 plasmids (Figure 2A) may have facilitated their transportation among different continents, which can also explain why IncI2 plasmids occupy the largest share of the *mcr-1*-bearing plasmids (41% of 229 *mcr-1*-bearing plasmids).

**FIGURE 3:**
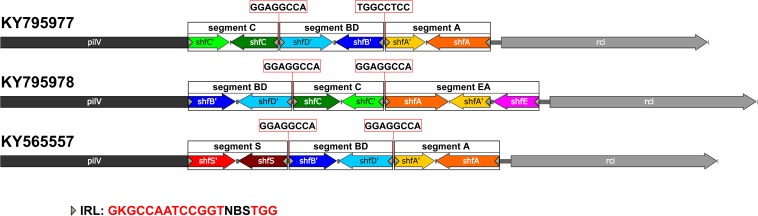
Typical shufflon structures in three representative IncI2 plasmids. Accession numbers is listed beside. The common inverted repeat sequence in shufflon segments is shown at the bottom as IRL. The highly conserved repeat (inverted or direct repeat) sequences between adjacent shufflon segments are shown in red frames.

The shufflons extracted from IncI2 plasmids were carefully annotated the structure. Typical shufflon structures are illustrated in Figure 3. There is a pair of inverted *sfx* repeats (IRL: **GKGCCAATCCGGT**NBS**TGG** and IRR**: CCA**SVN**ACCGGATTGGCMC**) at both ends of each shufflon segment, and also a highly conserved inverted or direct repeat sequence (GGAGGCCA) between adjacent shufflon segments. The shufflon structure also exhibits regional disparity (Supplementary Table S2), which is in good agreement with the clustering mode of IncI2 plasmids in the phylogenetic tree (Supplementary Figure S9B). IncI2-type plasmids isolated from Asia contain almost all kinds of shufflon structures. The shufflon rearrangement is closely related to plasmid transmission to a broad range of the Enterobacteriaceae (Ishiwa and Komano, 2003, 2004). This can explain why the most diverse *Enterobacteriaceae* hosts bearing *mcr-1*-positive IncI2 plasmids are detected in Asia (Figure 2A). The IncI2 plasmids isolated from Europe and North America commonly contain the shufflon segment E (except those plasmids with only one shufflon segment), while, to date (June 2018), no IncI2 plasmid from South America has been detected with definite structure of shufflon segment E (Supplementary Table S2). The *mcr-1*-bearing IncI2 plasmids in Europe and North America may mainly be derived from a plasmid with shufflon segment E, while those in South America may have been originated from one without the segment. Geographical barriers retain this original mark.

Some shufflon sequences cannot be annotated as accurate segmental structure, which could be due to sequencing/assembly mistakes (Supplementary Table S2). We also found an unnoticed shufflon segment structure that had never been annotated in the database. It is common and highly conserved in several IncI2 type plasmids (Supplementary Table S2, KY565557, CM008278, CP028153, CP006264, CP007134, FR851304, and CP030766), which cannot be accidental events. Thus we assigned the segment as segment S (Figure 3, KY565557).

The mechanism of Nested Russian Doll-like genetic mobility must be common for the worldwide dissemination of various mobilized ARGs (Dortet et al., 2014; Sheppard et al., 2016; Wang et al., 2018). The *bla*_N__DM_ and *bla*_KPC_ genes are mainly located on conjugative plasmids of several different incompatibility groups (Dortet et al., 2014; Sheppard et al., 2016), which is similar to the behavior of *mcr-1* gene. ARGs may be flanked by different MGE sequences at their mobilization, and exhibit different characteristics in their relocation process. The transfer of *mcr-1* is mediated by the insertion sequence IS*Apl1*. The high activity of this MGE enables *mcr-1* gene to jump flexibly between different plasmids and between different bacterial species (Supplementary Table S1). At bacterial genome level, no genotypic clustering by geographical origin and isolation source has been observed (Matamoros et al., 2017). While at the transposon level, scientists have predicted the time of the initial mobilization of *mcr-1* (Wang et al., 2018). In this work, at the plasmid level (one important genetic level involved in HGT) and according to the analysis routine illustrated in Supplementary Figure S1, we have found that the global spread of *mcr-1*-bearing plasmids is accompanied by their multiregional evolution. Based on the intrinsic mechanism of HGT, we believe that the analysis of mobilized ARGs at multiple levels of MGEs (transposons, integrons, and plasmids) can give important epidemiological information about their dissemination.

### Possible Intercontinental Transportation of Resistance Plasmids

**FIGURE 4:**
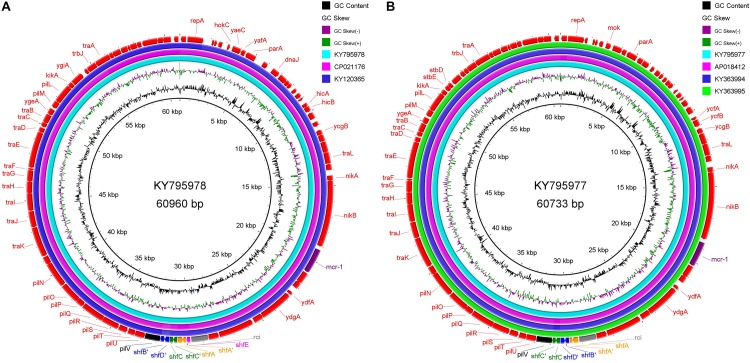
Sequence alignment of IncI2 plasmids with high homology, which were isolated from different regions along the East Asian-Australasian Flyway. **(A)** KY795978 is used as reference. **(B)** KY795977 as reference. The alignment was performed using BRIG tool (Alikhan et al., 2011). The outmost arrow ring signifies annotation of the reference sequence. The shufflon structure and the *mcr-1* gene are highlighted with outstanding colors.

Since we have found sequencing evidence for the horizontal transfer of ARGs between the environmental and avian bacteria, it is possible that migratory birds mediate the intercontinental transportation of the resistance plasmid. Two typical cases were noted at the analysis of IncI2 plasmids. First, the IncI2 plasmid, pJIE3685-1 (KY795978), isolated from Australia is of almost the same size (∼60960 bp) with two plasmids isolated from China (Taiwan), p5CRE51-MCR-1 (CP021176 from a human *E. coli*), and pP111 (KY120365, from a porcine *Salmonella enterica* subsp.). The position and the segment composition of the shufflon structure is also the same in the three plasmids (Supplementary Table S2), which indicates that the shufflon of the three plasmids is equivalent. Alignment of the remaining parts of the three plasmids after shufflon excision shows high identity among them with mismatches of only several (∼7 nts out of 59,042 nts) nucleotides (Figure 4A). In addition, another IncI2 plasmid from Australia, pJIE2288-1 (KY795977), is also highly identical (∼8 nts mismatches out of 59,081 nts) to the three plasmids from Asia (Figure 4B), pRYU2912C-1 (AP018412, Japan, from a *E. coli*), pSh113-m4 (KY363994, Shanghai, China, from a human *Shigella sonnei*) and pSh069-m6 (KY363995, Shanghai, China, from a human *Shigella sonnei*). Such close genetic relationships between these plasmids suggest that they do not seem to originate from a common evolutionary ancestor, but rather are duplicate offspring of the same plasmid. Two Australian plasmids are from *E. coli* strains that were isolated from two clinical patients at New South Wales. Neither patients had ever gone abroad or had taken colistin/polymyxin antimicrobial drugs during hospitalization (Ellem et al., 2017). We noticed that these highly homologous IncI2 plasmids were all found on the same flyway of migratory shorebirds, i.e., the East Asian-Australasian Flyway (Supplementary Figure S7). Many species of migratory shorebirds take extreme long-distance migration between eastern Russia and Oceania. During the migration, they stage once in Eastern Asia (typically the Yellow Sea region) for around 40 days, resting and refueling for the subsequent flight (Battley et al., 2012). There is continental-scale pollution of ARGs at the estuaries along the east coast of China, where the migratory shorebirds pass by Zhu et al. (2017). Migratory shorebirds can be an option for tracking the source of the resistance plasmids.

During the outbreak of avian influenza, migratory birds as potential global spreaders had attracted the attention of scientists (Liu et al., 2005; Normile, 2006; Olsen et al., 2006). However, according to a number of previously reported studies, the risk of migratory birds mediating intercontinental exchange of influenza virus, especially those highly pathogenic ones, is fairly low (Krauss et al., 2007; Langstaff et al., 2009). A sick bird can hardly accomplish the arduous task of long-distance (intercontinental) migration. Nevertheless, migratory shorebirds carrying drug-resistant symbiotic bacteria can cross the **Wallace Line** and bring ARGs from Asia to Australia (Supplementary Figure S7). Since the discovery of the mobilized colistin resistance gene, several studies have reported the detection of *mcr-1*-positive *E. coli* in migratory birds (Liakopoulos et al., 2016; Mohsin et al., 2016; Ruzauskas and Vaskeviciute, 2016; Sellera et al., 2017). Salt tolerant *mcr-1*-positive *E. coli* strains have also been isolated from recreational waters of public urban beaches (Fernandes et al., 2017). Therefore, *mcr-1*-bearing plasmids may be transported, via avian migration, from Eastern Asia to Australia, where the usage of antibiotics is under strict control but is definitely affected by migratory shorebirds. In fact, *mcr-1*-positive *E. coli* has recently been isolated from wild bird (silver gull) in Australia (Mukerji et al., 2019). In view of limited data available for bacterial ARGs of migratory birds, it is difficult to discuss the global dissemination of ARGs in the context of avian ecology. Our analysis does not imply that avian migration is the main route for the global dissemination of antibiotic resistance. In today’s highly globalized world, global population mobility and international trade, especially the trade of food animal, must be the main channels (Matamoros et al., 2017; Shen et al., 2018; Hernando-Amado et al., 2019). Even so, the large number of migratory birds and their fixed migration path will have a lasting impact on the receptor environment. In particular, different countries execute different strategies for using antibiotics. Antibiotics used for human in one country may be used as animal feed additives in another. If migratory birds transport antibiotic resistance from the latter environment to the former, the cause originated in one country may have a devastating consequence in another. Nowadays, with the global dissemination of antibiotic resistance, no country can be immune from it. The application of antibiotics should follow the strategy of global unification.

## Data Availability Statement

Publicly available datasets were analyzed in this study. This data can be found in GenBank database under the accession numbers CP032986 to CP032995.

## Author Contributions

KY and JW designed the study. DR isolated the *E. coli* strains and evaluated their drug resistance. JW and YL prepared the DNA samples for sequencing and analyzed the sequencing data. XD and LZ performed the metadata analysis of the *mcr-1*-bearing plasmids. YL, JW, and KY constructed the manuscript. YF edited the manuscript and put forward constructive suggestions on it. All authors reviewed, revised, and approved the final report.

## Conflict of Interest

The authors declare that the research was conducted in the absence of any commercial or financial relationships that could be construed as a potential conflict of interest.

## Footnotes

1 https://cge.cbs.dtu.dk/services/MLST/

2 https://cge.cbs.dtu.dk/services/PlasmidFinder/

3 https://cge.cbs.dtu.dk/services/ResFinder/

4 https://mafft.cbrc.jp/alignment/server/
