# Supplementary material for: Metadata Analysis of mcr-1-Bearing Plasmids Inspired by the Sequencing Evidence for Horizontal Transfer of Antibiotic Resistance Genes Between Polluted River and Wild Birds
Source: Front Microbiol. 2020 Mar 10;11:352. doi: 10.3389/fmicb.2020.00352 (PMC7076156; doi:10.3389/fmicb.2020.00352)
Supplement: Supplementary file 1 [file Data_Sheet_1.docx]

**Supplementary information for:**

**Title: Metadata analysis of *mcr-1*-bearing plasmids inspired by the sequencing evidence for horizontal transfer of antibiotic resistance genes between polluted river and wild birds**

**Authors:** Yufei Lin^1,#^, Xiaohong Dong^1,#^, Jiao Wu^1,#^, Dawei Rao^1^, Lihua Zhang^1^ Yousef Faraj^1^ and Kun Yang^1,^*

**Affiliation:** ^1^Department of Pharmaceutical & Biological Engineering, School of Chemical Engineering, Sichuan University, Chengdu, China.

^#^These authors contributed equally: Yufei Lin, Xiaohong Dong and Jiao Wu.

***Correspondence to:**

Kun Yang

[cookyoung@scu.edu.cn](mailto:cookyoung@scu.edu.cn)

**Supplementary Information Table of Contents**

| **Supplementary Figures** |  |
| --- | --- |
| Supplementary Figure S1. Diagram of the analysis routine of *mcr-1*-bearing plasmids. | 3 |
| Supplementary Figure S2. Gene components of three *E. coli* isolates. | 4 |
| Supplementary Figure S3. Sequence alignment of *mcr-1*-containing segments on *mcr-1*-bearing plasmids of the three *E. coli* isolates. | 5 |
| Supplementary Figure S4. Sequencing evidence for horizontal transfer of ARGs between the environmental (polluted Jin River) and avian (egret) *E. coli*. | 6-8 |
| Supplementary Figure S5. The same drug-resistance transposon occurring on both chromosome (W5-6Chr) and plasmids (pMCR_W5-6 and p2_BE2-5) among different host strains. | 9 |
| Supplementary Figure S6. Distribution of ARGs and MGEs on plasmids (A) p2_W5-6, (B) p3_W5-6, (C) pMCR_W2-5, (D) p2_W2-5 and (E) pMCR_BE2-5. | 10 |
| Supplementary Figure S7. Global geographical distribution of incompatibility groups of *mcr-1*-bearing plasmids. | 11 |
| Supplementary Figure S8. Phylogenetic relationship among IncX4 plasmids. | 12-13 |
| Supplementary Figure S9. Phylogenetic relationship among IncI2 plasmids (A) and the shufflon structures on these plasmids (B). | 14-15 |
| **Supplementary Tables** |  |
| Supplementary Table S1. Plasmid metadata. | 16 |
| Supplementary Table S2. Shufflon structure of IncI2 plasmids. | 17 |
| **Supplementary References** | 18 |

**
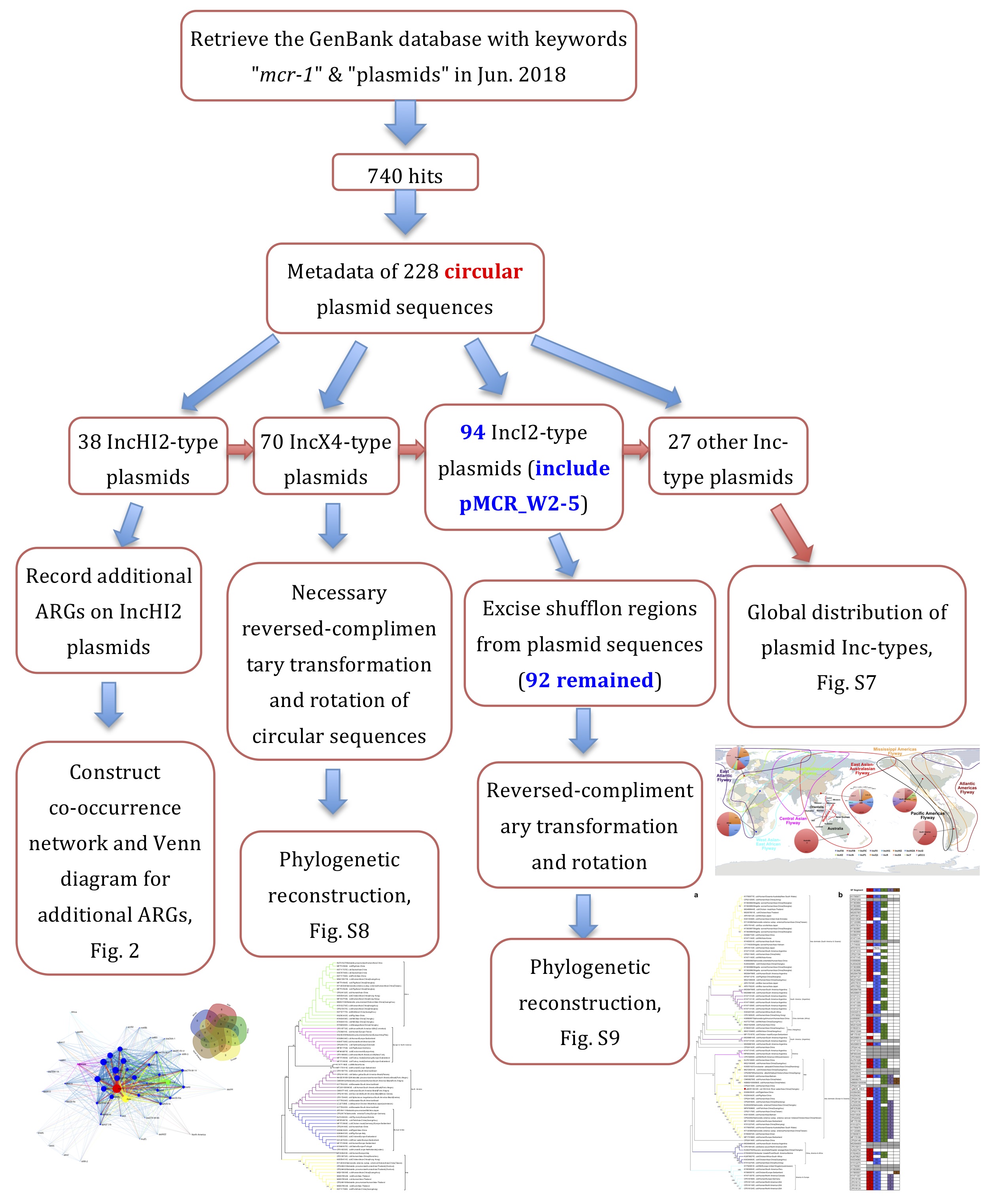
**

**Supplementary Figure S1. Diagram of the analysis routine of *mcr-1*-bearing plasmids.** Briefly, the global distribution of various plasmid types was visualized in a world map. The targeted analysis was performed against the most abundant three types of plasmids according to their respective characteristics. For IncHI2-type plasmids that harbor the most various ARGs, we analyzed the correlation between these ARGs and their geographical distribution via co-occurrence network analysis (performed in Matlab_R2016a). For the highly conserved InX4-type plasmids, we performed the phylogenetic reconstruction in MEGA7 (Kumar et al., 2016) based on sequence alignment. The sequence alignment was executed with a multiple sequence alignment program MAFFT (Katoh et al., 2017). For IncI2-type plasmids, shufflon regions of high frequency recombination were excised from plasmid sequences, and then the sequences after shufflon removal were used for alignment and phylogenetic reconstruction.


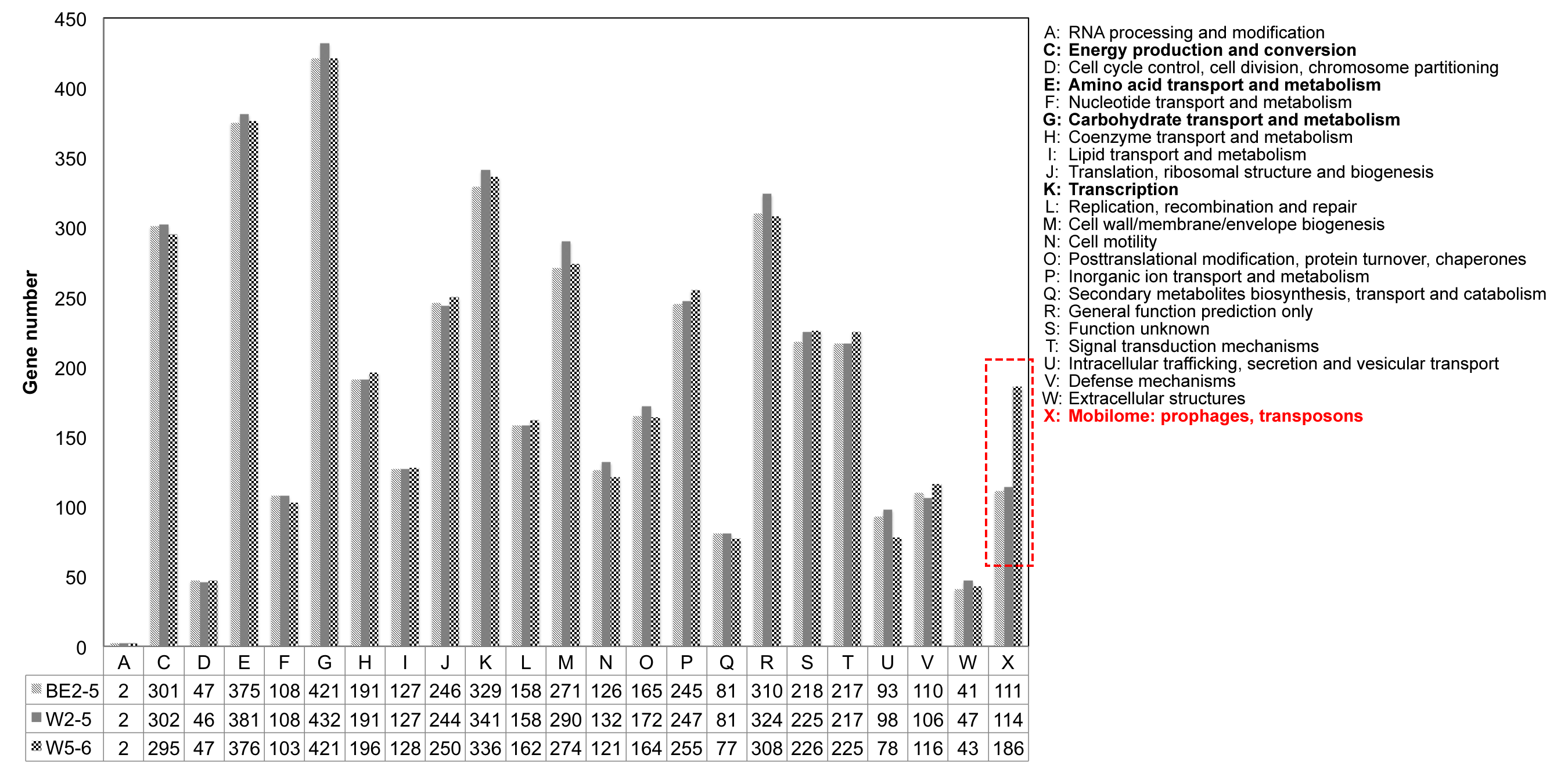


**Supplementary Figure S2. Gene components of three *E. coli* isolates.** The red rectangular dot-line frame indicates the high composition of mobilome in W5-6.


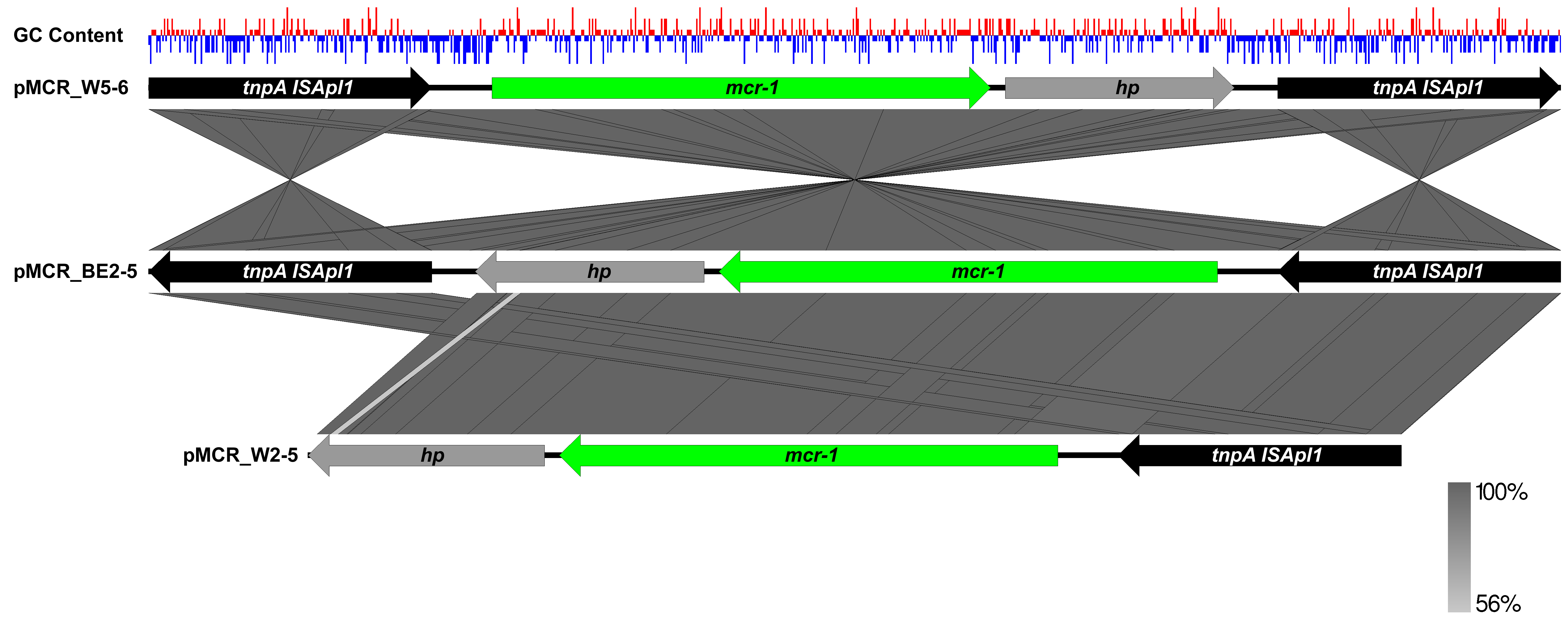


**Supplementary Figure S3. Sequence alignment of *mcr-1-*containing segments on *mcr-1*-bearing plasmids of the three *E. coli* isolates.** Transposase genes (*tnpA* IS*Apl1*) are indicted with black arrows. The *mcr-1*-containing segment on plasmid pMCR_W2-5 lost the downstream IS*Apl1*.

**
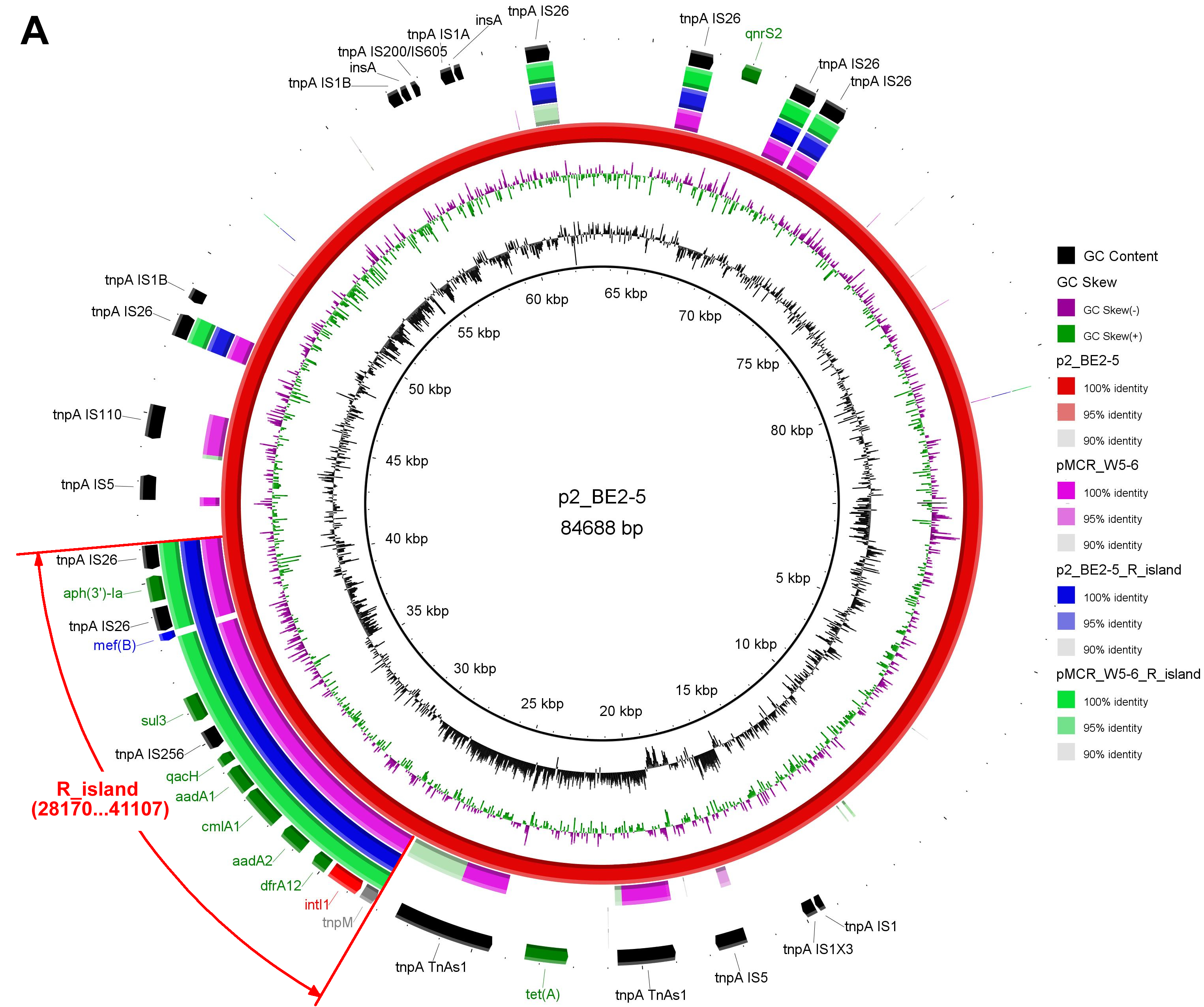
**

**
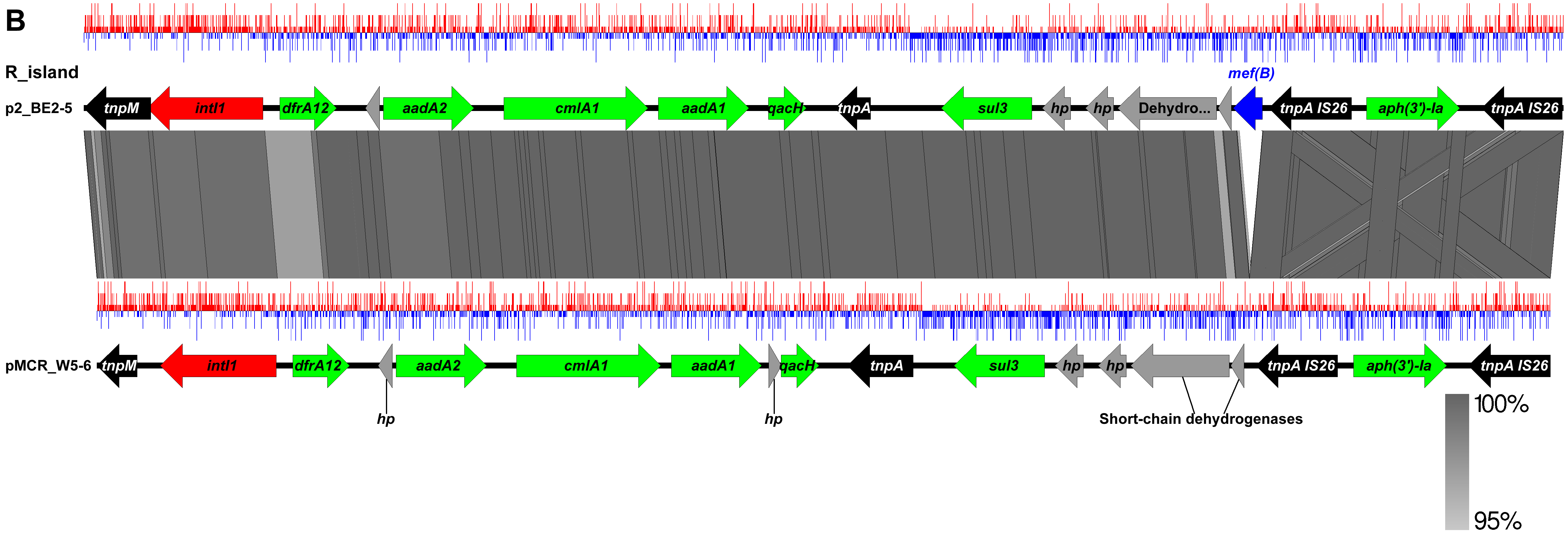
**

**Supplementary Figure S4. Sequencing evidence for horizontal transfer of ARGs between the environmental (polluted Jin River) and avian (egret) *E. coli*.** Panel (A) exhibits an alignment for the two bacterial plasmids of environmental- or avian-origin constructed with BRIG tool(Alikhan et al., 2011), which indicates that highly homologous MDR regions (R_island) are detected on the plasmids of different Inc-types and of different origination. The sequence of the plasmid p2_BE 2-5 is used as reference sequence. The sequences of plasmid pMCR_W5-6, MDR regions on the environment-originated (pMCR_W5-6_R_island) and egret-originated (p2_BE2-5_R_island) plasmids are incorporated into the alignment. It should be noticed that BRIG tool only displays the homologous sequences on different plasmids without giving the location information of these sequences on query plasmids. ARGs and MGEs are annotated to the reference sequence. Easyfig(Sullivan et al., 2011) constructed alignment of the sequences of the MDR regions on these two plasmids is shown in panel (B). The GC content and the arrangement of ARGs (green arrows), genes of transposon-related enzymes (black arrows), the integrase gene *intI1* (red arrows) and other coding-sequences (grey arrows) in the MDR regions are illustrated in this figure. The *mef(B)* gene missing from the plasmid pMCR_W5-6 is highlighted in blue. The annotation files of the MDR regions submitted to the Easyfig tool are generated with the prokaryote genome annotation tool RASTtk(Brettin et al., 2015).


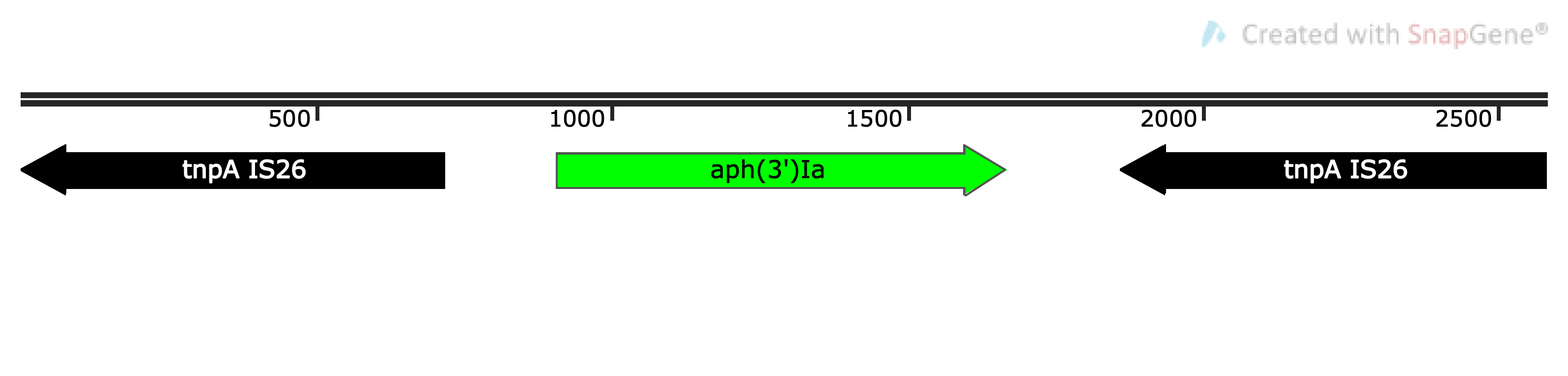


**Supplementary Figure S5. The same drug-resistance transposon occurring on both chromosome (W5-6Chr) and plasmids (pMCR_W5-6 and p2_BE2-5) among different host strains.**


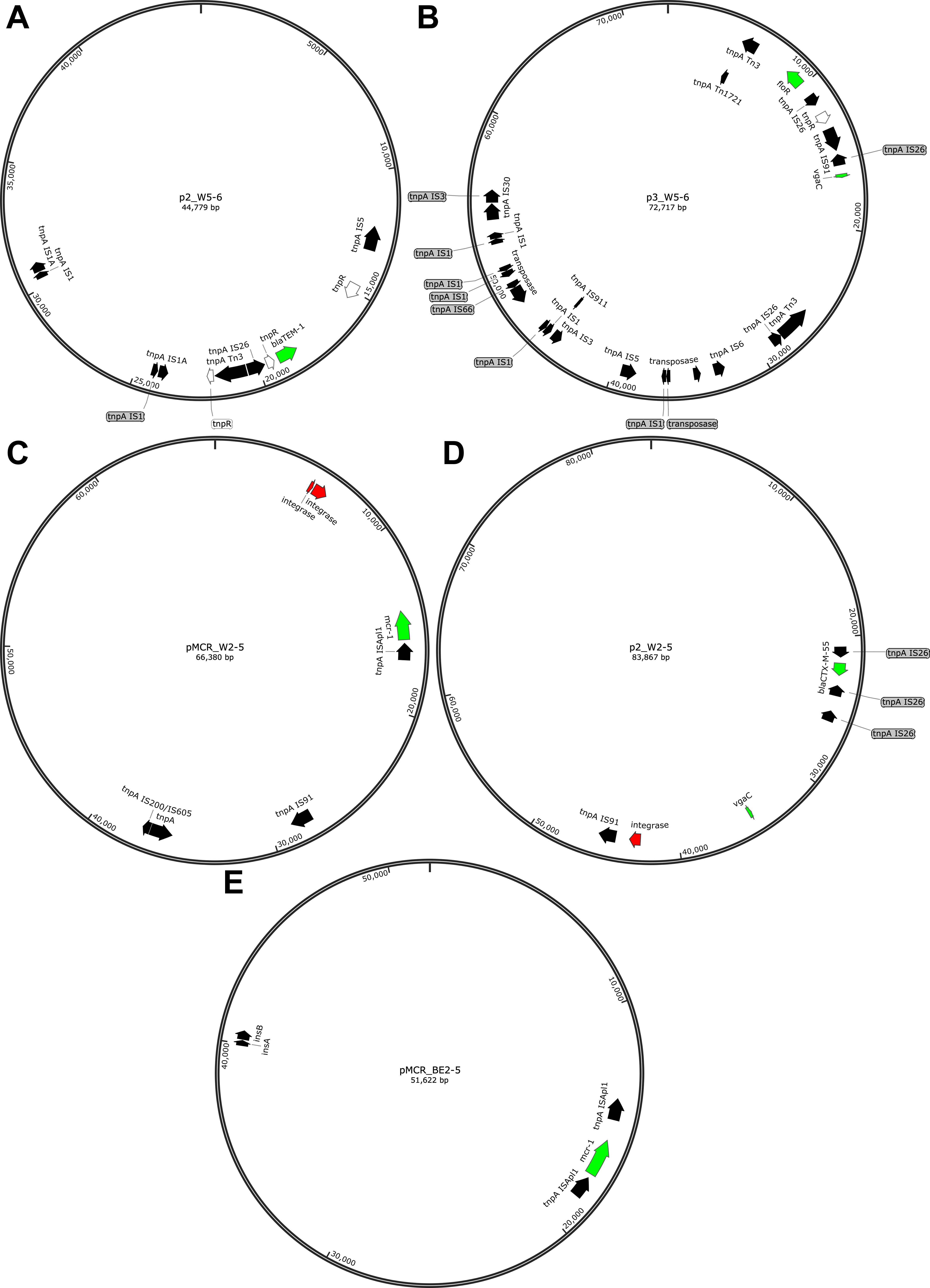


**Supplementary Figure S6. Distribution of ARGs (green arrows) and MGEs (black arrows for transposase genes and red arrows for site-specific integrase/recombinase genes) on plasmids (A) p2_W5-6, (B) p3_W5-6, (C) pMCR_W2-5, (D) p2_W2-5 and (E) pMCR_BE2-5.**


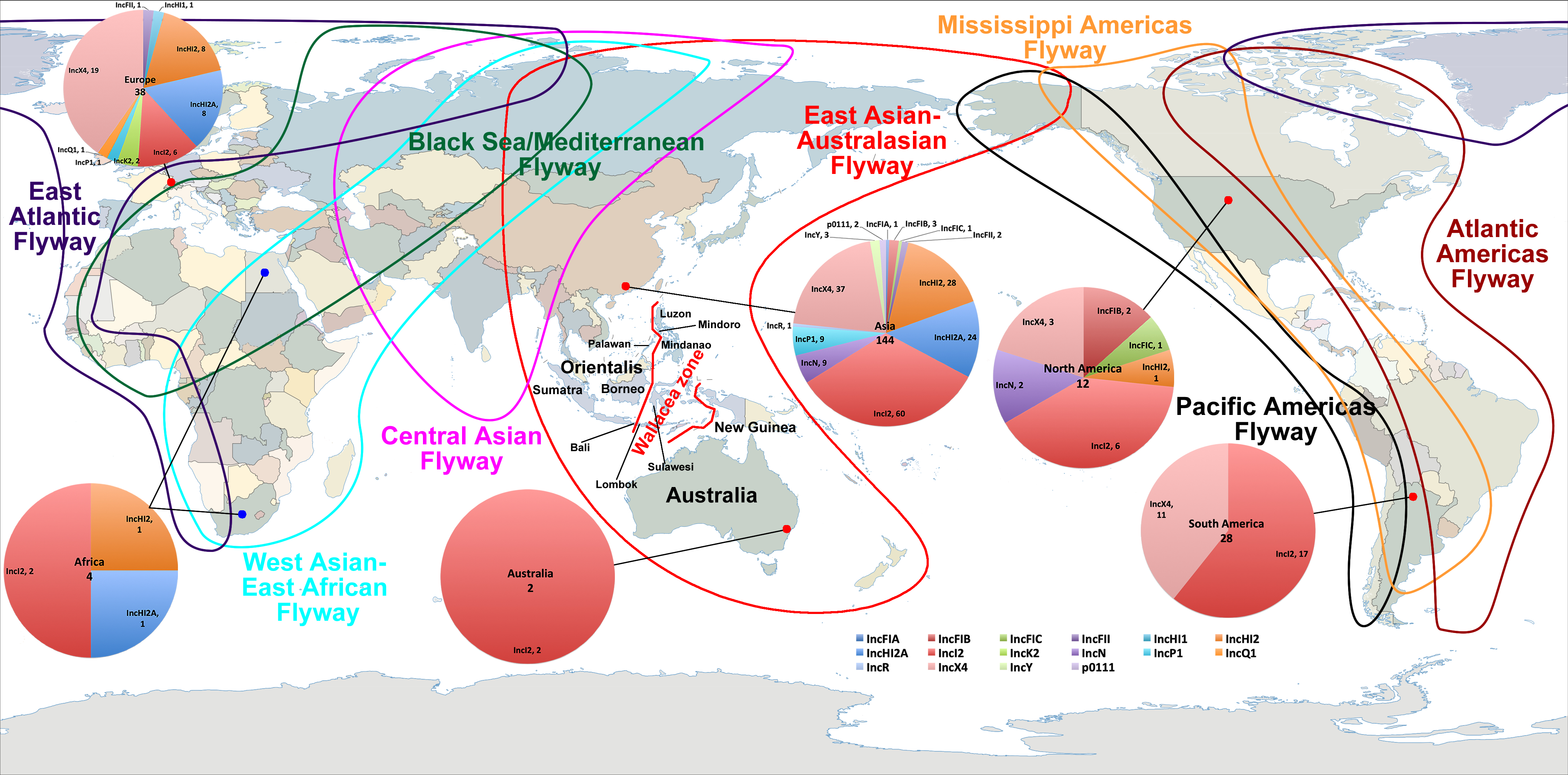


**Supplementary Figure S7.** **Global geographical distribution of incompatibility groups of *mcr-1*-bearing plasmids.** The figure is modified from a world map (map number GS20161663) downloaded from the open source website, http://bzdt.ch.mnr.gov.cn. Simplified global main flyways of migratory birds are labeled on the map(Olsen et al., 2006). The Wallacea zone is plotted according to the information provided on Wikipedia (https://wiki2.org/en/Wallace_line). A total of 38 IncHI2, 70 IncX4, 94 IncI2 and 29 other Inc-type plasmids are incorporated in this analysis. The numbers of plasmids isolated from different regions are listed in pie charts.

**Supplementary Figure S8. Phylogenetic relationship among IncX4 plasmids.** The evolutionary history was inferred using the Neighbor-Joining method(Saitou and Nei, 1987). The optimal tree with the sum of branch length = 0.01437344 is shown. The percentages of replicate trees in which the associated nodes clustered together in the bootstrap test (500 replicates) are shown next to the branches(Felsenstein, 1985). The evolutionary distances were computed using the Maximum Composite Likelihood method(Tamura et al., 2004) and were in the units of the number of base substitutions per site. The analysis involved 70 nucleotide sequences. All positions containing gaps and missing data were eliminated. There were a total of 27,254 positions in the final dataset. Evolutionary analyses were conducted in MEGA7(Kumar et al., 2016).


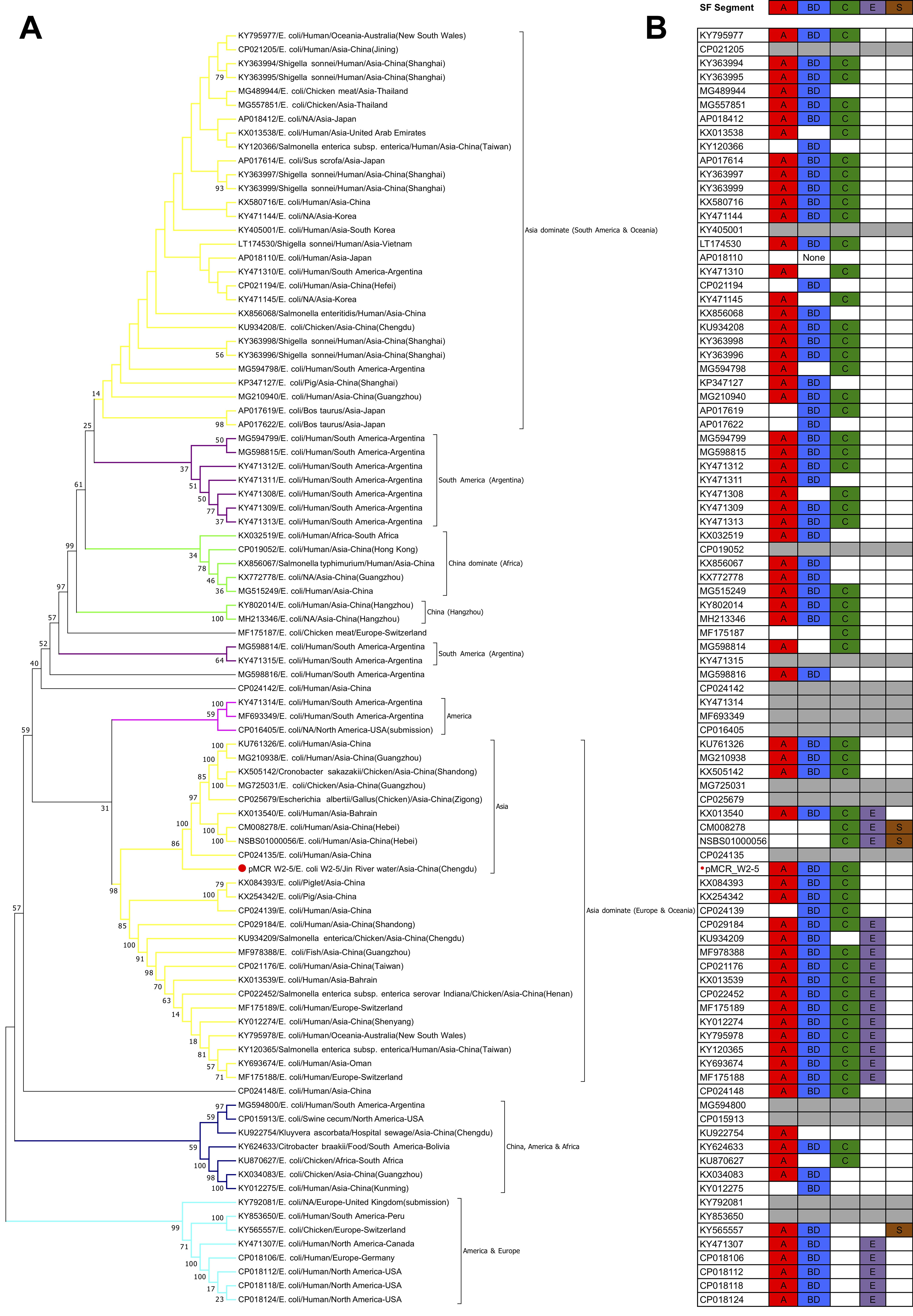


**Supplementary Figure S9. Phylogenetic relationship among IncI2 plasmids (A) and the shufflon structures on these plasmids (B).** (**A**): The phylogenetic reconstruction was based on the alignment of IncI2 plasmid sequences that were excised the shufflon sequence (a high recombination region), respectively. The evolutionary history was inferred using the Neighbor-Joining method(Saitou and Nei, 1987). The optimal tree with the sum of branch length = 0.18907576 is shown. The percentages of replicate trees in which the associated nodes clustered together in the bootstrap test (500 replicates) are shown next to the branches(Felsenstein, 1985). The evolutionary distances were computed using the Maximum Composite Likelihood method(Tamura et al., 2004) and were in the units of the number of base substitutions per site. The analysis involved 92 nucleotide sequences. All positions containing gaps and missing data were eliminated. There were a total of 41,295 positions in the final dataset. Evolutionary analyses were conducted in MEGA7(Kumar et al., 2016). The plasmid pMCR_W2-5 isolated in this work is labeled with red dot. (**B**): The shufflon (SF) segment compositions of IncI2 plasmids are listed. The grey ones indicate those plasmids whose shufflon region cannot be accurately annotated due to possible sequencing/assembly mistakes. The plasmids from Europe and North America commonly contain the shufflon segment E, while the plasmids from South America do not have this segment.

**Supplementary Table S1. Metadata of *mcr-1*-bearing plasmids.**

Provided in a separate excel spreadsheet.

**Supplementary Table S2. Shufflon structures of IncI2 plasmids.**

Provided in a separate excel spreadsheet.

**Supplementary References**

Alikhan, N.F., Petty, N.K., Ben Zakour, N.L., and Beatson, S.A. (2011). BLAST Ring Image Generator (BRIG): simple prokaryote genome comparisons. *Bmc Genomics* 12. doi: 10.1186/1471-2164-12-402.

Brettin, T., Davis, J.J., Disz, T., Edwards, R.A., Gerdes, S., Olsen, G.J., et al. (2015). RASTtk: a modular and extensible implementation of the RAST algorithm for building custom annotation pipelines and annotating batches of genomes. *Sci Rep* 5**,** 8365. doi: 10.1038/srep08365.

Felsenstein, J. (1985). Confidence limits on phylogenies: an approach using the bootstrap. *Evolution* 39(4)**,** 783-791. doi: 10.1111/j.1558-5646.1985.tb00420.x.

Katoh, K., Rozewicki, J., and Yamada, K.D. (2017). MAFFT online service: multiple sequence alignment, interactive sequence choice and visualization. *Brief Bioinform*. doi: 10.1093/bib/bbx108.

Kumar, S., Stecher, G., and Tamura, K. (2016). MEGA7: Molecular Evolutionary Genetics Analysis version 7.0 for bigger datasets. *Mol Biol Evol* 33(7)**,** 1870-1874. doi: 10.1093/molbev/msw054.

Olsen, B., Munster, V.J., Wallensten, A., Waldenstrom, J., Osterhaus, A.D., and Fouchier, R.A. (2006). Global patterns of influenza a virus in wild birds. *Science* 312(5772)**,** 384-388. doi: 10.1126/science.1122438.

Saitou, N., and Nei, M. (1987). The neighbor-joining method: a new method for reconstructing phylogenetic trees. *Mol Biol Evol* 4(4)**,** 406-425. doi: 10.1093/oxfordjournals.molbev.a040454.

Sullivan, M.J., Petty, N.K., and Beatson, S.A. (2011). Easyfig: a genome comparison visualizer. *Bioinformatics* 27(7)**,** 1009-1010. doi: 10.1093/bioinformatics/btr039.

Tamura, K., Nei, M., and Kumar, S. (2004). Prospects for inferring very large phylogenies by using the neighbor-joining method. *Proc Natl Acad Sci U S A* 101(30)**,** 11030-11035. doi: 10.1073/pnas.0404206101.
